# Two‐Dimensional Bis(dithiolene)iron(II) Self‐Powered UV Photodetectors with Ultrahigh Air Stability

**DOI:** 10.1002/advs.202100564

**Published:** 2021-05-19

**Authors:** Ying‐Chiao Wang, Chun‐Hao Chiang, Chi‐Ming Chang, Hiroaki Maeda, Naoya Fukui, I‐Ta Wang, Cheng‐Yen Wen, Kuan‐Cheng Lu, Shao‐Ku Huang, Wen‐Bin Jian, Chun‐Wei Chen, Kazuhito Tsukagoshi, Hiroshi Nishihara

**Affiliations:** ^1^ International Center for Young Scientists (ICYS) and WPI International Center for Materials Nanoarchitectonics (WPI‐MANA) National Institute for Materials Science (NIMS) Tsukuba Ibaraki 305‐0044 Japan; ^2^ Department of Materials Science and Engineering National Taiwan University Taipei 10617 Taiwan; ^3^ Department of Electrophysics National Chiao Tung University Hsinchu 30010 Taiwan; ^4^ Department of Chemistry School of Science The University of Tokyo Tokyo 113‐0033 Japan; ^5^ Research Center for Science and Technology Tokyo University of Science Chiba 278‐8510 Japan; ^6^ Center of Atomic Initiative for New Materials (AI‐MAT) National Taiwan University Taipei 10617 Taiwan

**Keywords:** iron, metal‐organic frameworks (MOFs), self‐powered UV photodetectors, ultrahigh air stability, *π*‐conjugated coordination nanosheets

## Abstract

Organometallic two‐dimensional (2D) nanosheets with tailorable components have recently fascinated the optoelectronic communities due to their solution‐processable nature. However, the poor stability of organic molecules may hinder their practical application in photovoltaic devices. Instead of conventional organometallic 2D nanosheets with low weatherability, an air‐stable *π*‐conjugated 2D bis(dithiolene)iron(II) (FeBHT) coordination nanosheet (CONASH) is synthesized via bottom‐up liquid/liquid interfacial polymerization using benzenehexathiol (BHT) and iron(II) ammonium sulfate [Fe(NH_4_)_2_(SO_4_)_2_] as precursors. The uncoordinated thiol groups in FeBHT are easily oxidized, but the Fe(NH_4_)_2_(SO_4_)_2_ dissociation rate is slow, which facilitates the protection of sulfur groups by iron(II) ions. The density functional theory calculates that the resultant FeBHT network gains the oxygen‐repelling function for oxidation suppression. In air, the FeBHT CONASH exhibits self‐powered photoresponses with short response times (<40 ms) and a spectral responsivity of 6.57 mA W^−1^, a specific detectivity of 3.13 × 10^11^ Jones and an external quantum efficiency of 2.23% under 365 nm illumination. Interestingly, the FeBHT self‐powered photodetector reveals extremely high long‐term air stability, maintaining over 94% of its initial photocurrent after aging for 60 days without encapsulation. These results open the prospect of using organometallic 2D materials in commercialized optoelectronic fields.

## Graphical abstract

The organometallic bis(dithiolene)iron(II) (FeBHT) 2D coordination nanosheets (CONASHs) are used to fabricate self‐powered photodetectors, resulting in a superior UV photoresponse. The effective suppression of oxygen molecule adsorption on FeBHT CONASHs is responsible for the device stability in air. This function provides guidance for the design of unstable organic–inorganic 2D hybrid nanomaterials in practical optoelectronic devices.

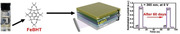

## 1 Introduction

Two‐dimensional (2D) organic‐inorganic hybrid nanosheets consisting of atomic, molecular, and ionic bonds have attracted unprecedented attention owing to their outstanding features.^[^ ^1^, ^2^, ^3^, ^4^, ^5^ ^]^ Coordination nanosheets (CONASHs), which are composed of tiled organic ligand molecules with inorganic metal ions in a 2D network, are a particularly attractive alternative.^[^ ^6^, ^7^, ^8^, ^9^, ^10^ ^]^ With bottom‐up self‐assembly approaches, such as electrocatalysis^[^ ^11^, ^12^ ^]^ and photonics,^[^ ^13^ ^]^ various possible combinations of organic anions and inorganic cations allow the CONASH properties to be adjusted. With the advantages of its redox nature and light sensitivity, a bis(dipyrrinato)zinc(II) CONASH containing the dipyrrin ligand (**L1**) was first reported as a photoabsorber in a liquid electrolyte‐filled photoelectrochemical cell, which generated a quantum yield (*φ*) of 0.86% for photoelectric conversion.^[^ ^14^ ^]^ After replacing molecule **L1** with a porphyrin‐dipyrrin hybrid ligand (**L2**), the porphyrin unit with an enhanced photoresponse nature resulted in a widened absorption band from the region of 450–550 nm to 400–650 nm and an enhanced *φ* of 2.02%.^[^ ^15^ ^]^ However, these photoelectrochemical cells generally suffer from durability issues (e.g., electrolyte leakage, CONASH corrosion, and electrode destruction) due to their adoption of liquid electrolytes;^[^ ^16^, ^17^ ^]^ this issue may need to be solved to realize the practical application of CONASH‐based photoelectric conversion systems.

To overcome the shortcomings of liquid electrolytes, the ultimate strategy will consist of all solid‐state architectures. Typically, when introducing light‐harvesters into solid‐state electronic devices, the electrical conductivity rather than the ionic conductivity should be considered. Unfortunately, zinc(II)‐based CONASHs are regarded as electrical insulators because the overlap between the d‐orbitals of zinc(II) ions and the *π*‐orbitals of ligands is small (because of the lack of empty d‐orbitals in zinc(II) ions). Instead of zinc, iron is the most promising candidate since there are empty d‐orbitals in iron(II) ions, and more importantly, because of its abundance in the Earth's crust.^[^ ^18^ ^]^ A recent advancement was the use of an Fe_3_(THT)_2_(NH_4_)_3_ (THT = 2,3,6,7,10,11‐triphenylenehexathiol) film in solid‐state photodetectors, which exhibited a maximum spectral responsivity (R) of 4 mA W^−1^.^[^ ^19^ ^]^ Nevertheless, charge carrier transport paths may be obstructed due to the presence of large ligands (e.g., molecules **L1**, **L2**, and THT) in the CONASHs, resulting in longer distances between the metal centers^[^ ^20^ ^]^ or unevenness CONASHs^[^ ^21^, ^22^ ^]^ (e.g., CONASHs prepared using nonplanar soft ligands **L1** and **L2**), consequently limiting their photoelectric conversion efficiencies.

**Figure 1:**
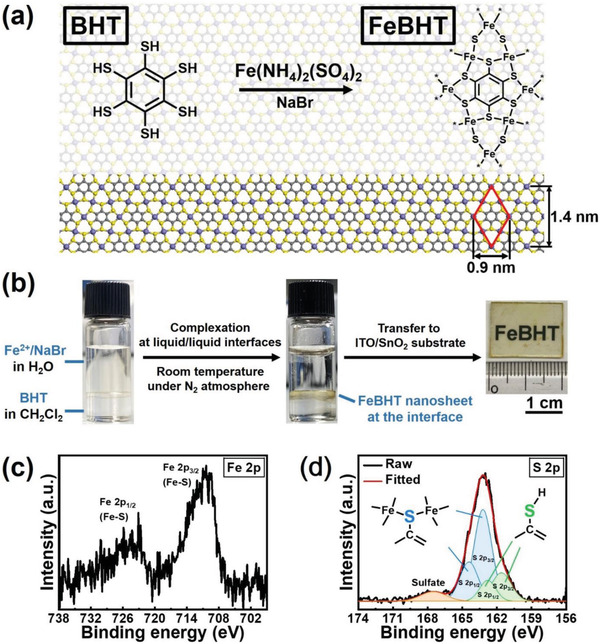
The formation and material characterization of FeBHT nanosheets. a) Chemical structure of the BHT ligand and the resultant FeBHT complex. b) Photographs of the liquid/liquid interfacial synthesis process before and after the formation of the FeBHT film, and a representative photograph of an FeBHT nanosheet that was transferred to an ITO glass/SnO_2_ substrate. High‐resolution XPS of the c) Fe 2*p* and d) S 2*p* core levels of the FeBHT nanosheet.

Motivated by the above requirements, we designed a bottom‐up 2D *π*‐conjugated CONASH comprising a bis(dithiolene)iron(II) complex [FeBHT in **Figure** **1a**; this chemical structure could be determined from the X‐ray diffraction (XRD) analysis shown in Figure S1 (Supporting Information) and could be further confirmed by our previous XRD result]^[^ ^23^ ^]^ to act as the photoactive layer in a photodetector with a structure containing indium tin oxide (ITO)/SnO_2_/FeBHT/2,2′,7,7′‐tetrakis‐(*N*,*N*‐di‐*p*‐methoxyphenylamine)9,9′‐spirobifluorene (Spiro‐OMeTAD)/Au, wherein we replaced the liquid electrolytes with a Spiro‐OMeTAD solid‐state layer (known as an efficient hole transporter^[^ ^24^, ^25^, ^26^ ^]^) to form a liquid‐free device. In addition, FeBHT was synthesized via coordination reactions between iron(II) ammonium sulfate [Fe(NH_4_)_2_(SO_4_)_2_] and benzenehexathiol (BHT). Among them, BHT is a small, planar and rigid ligand; hence, several CONASHs containing the BHT ligand have presented excellent electrical conductivity due to their good coplanarities, as well as the metal–metal distance‐shortening effect.^[^ ^20^, ^27^, ^28^, ^29^, ^30^ ^]^ Combining these material characteristics, we show that the FeBHT photodetectors had dramatically enhanced photoresponses, with a *φ* value of 5.94%, an R of 6.57 mA W^−1^, specific detectivity of 3.13 × 10^11^ Jones, external quantum efficiency of 2.23% and short response times (<40 ms) under 365 nm illumination at zero bias. We found that the dissociation rate of the Fe(NH_4_)_2_(SO_4_)_2_ salt is relatively slow, which promotes high‐aspect‐ratio film growth as a stabilizing effect, consistent with the density functional theory (DFT) calculations. In addition to its good durability, the unencapsulated FeBHT device exhibited the highest air stability reported so far for self‐powered photodetectors equipped with organic–inorganic hybrid photoabsorbers (Figure S2a, Supporting Information)^[^ ^31^, ^32^, ^33^ ^]^ and was comparable to all‐inorganic systems (Figure S2b, Supporting Information);^[^ ^34^, ^35^, ^36^, ^37^, ^38^, ^39^ ^]^ 94% of the photocurrent is retained after 60 days of exposure to air, which implies that the organometallic 2D nanosheets used in the laboratory research can be used in actual optoelectronic applications.

## 2 Results

### 2.1 Synthesis of FeBHT CONASHs

Figure 1b depicts the liquid/liquid biphasic synthesis of FeBHT CONASHs via a Fe^2+^ and NaBr‐containing aqueous solution (upper layer) and BHT‐dissolved dichloromethane (DCM, that is, CH_2_Cl_2_) (lower layer). The detailed processes can be found in the Experimental Section. After these two phases were layered, the initial color of the interface, which was transparent, spontaneously turned opaque upon the formation of CONASHs. Note that the source of the Fe^2+^ ions clearly affects the FeBHT film formation. Two representative iron salts of Fe(NH_4_)_2_(SO_4_)_2_ and Fe(CH_3_COO)_2_ were used to fabricate the FeBHT films. The as‐prepared FeBHT synthesized from Fe(NH_4_)_2_(SO_4_)_2_ (Figure S3a, Supporting Information) has a significantly reduced number of cracks compared to Fe(CH_3_COO)_2_ (Figure S3b, Supporting Information). As the salt gradually dissolves (Figure S4, Supporting Information), Fe(NH_4_)_2_(SO_4_)_2_ can ensure the slow and controllable coordination of Fe^2+^ ions with BHT. Therefore, tailored salts can effectively order the internal structures of CONASHs.

### 2.2 Component Analysis

The resultant FeBHT was insoluble in both water and DCM, and thus, it could be deposited easily on substrates at the bottom of the reactor. We then transferred the FeBHT nanosheet onto the ITO/SnO_2_ substrate and washed it with ethanol, where ITO and SnO_2_ functioned as anodes and electron transporters in FeBHT photodetectors, respectively. In particular, the transferred light‐green FeBHT CONASH on the ITO/SnO_2_ substrate has a centimeter‐scale semitransparent appearance. Furthermore, the bonding properties inside the FeBHT CONASH could be further studied by X‐ray photoelectron spectroscopy (XPS). As presented in Figure 1c, the typical dual binding energies of Fe 2p located at 710.8 and 724.3 eV were ascribed to the existence of the Fe—S bond,^[^ ^40^ ^]^ implying that the thiol terminal groups of BHT bonded with Fe^2+^ ions. Similarly, the S 2p peaks in the XPS spectrum at 159 and 166 eV could be split into two pairs of doublet peaks, assignable to the sulfur atoms that appeared in the FeBHT framework (163.2 eV for S 2p_3/2_ and 164.4 eV for S 2p_1/2_) and the sulfur atoms from BHT (161.6 eV for S 2p_3/2_ and 162.8 eV for S 2p_1/2_),^[^ ^41^ ^]^ as shown in Figure 1d. The newly generated S—Fe bond is consistent with that of the coordination of the BHT moiety with Fe^2+^ ions in the bis(dithiolene)iron(II) complex. The remaining BHT signals may come from the grain boundaries of FeBHT or unreacted BHT molecules. In addition, a minor broad peak position located at 167.8 eV was assigned to sulfate^[^ ^42^ ^]^ in the FeBHT nanosheet due to the presence of trace reaction residues. The surface morphology of the FeBHT complex was subsequently investigated by transmission electron microscopy (TEM). Figure S5a–e (Supporting Information) displays the TEM image, scanning TEM high‐angle annular dark field (STEM HAADF) image and the associated energy dispersive X‐ray spectroscopy (EDX) elemental maps (S, Fe, and C) of the FeBHT CONASH. Among these images, Figure S5d (Supporting Information) revealed that Fe elements are uniformly incorporated into the FeBHT framework. In addition, the TEM image of FeBHT showed some dark‐colored rods dispersed on the flat surface (Figure S5a, Supporting Information). According to the iron element distribution in the FeBHT framework (Figure S5d, Supporting Information), the rods contained a large number of Fe elements. Hence, there is a considerable probability that the sulfate peak positioned at 167.8 eV in the S 2p XPS spectrum (Figure 1d) comes from Fe(NH_4_)_2_(SO_4_)_2_ residues. These results demonstrate that the FeBHT film has a high‐aspect‐ratio sheet‐like architecture with the proposed compositions.

### 2.3 Electrical Conductivity

Prior to incorporating the light characteristics of the FeBHT complex into optoelectronic devices, its electrical conductivity evolution was first elucidated. To promote electronic communication within CONASHs, the chelating ligand needs to provide a favorable electronic coupling pathway. A more coplanar geometry of homobimetallic organometallic complexes and reduced metal distances in the CONASH network promote its intramolecular electron transfer.^[^ ^43^, ^44^ ^]^ Herein, BHT (Figure S6a, Supporting Information) and the control *π*‐conjugated ligand [2,2′:6′,2″‐terpyridine (TPY) (Figure S6b, Supporting Information)] were used to prepare FeBHT and FeTPY, respectively, to illustrate that the electrical conductivities of iron‐containing CONASHs may be changed by various ligands. As a result, a significant increase in the conductivity of the FeBHT film was found (Figure S6c, Supporting Information). The presence of almost planar BHT moieties that are smaller in size than TPY inhibits the rotation of the FeBHT network and minimizes the distance between metals, thereby modulating its electronic communication. By contrast, the single bond between two aromatic rings in the large‐sized TPY molecule is rotatable, resulting in poor coplanarity, longer metal distances and low electrical conductivity of the resultant FeTPY. Hence, through a reasonable design of organic ligands, the carrier transport within the CONASH framework can be substantially improved.

### 2.4 FeBHT Photodetector Design

**Figure 2:**
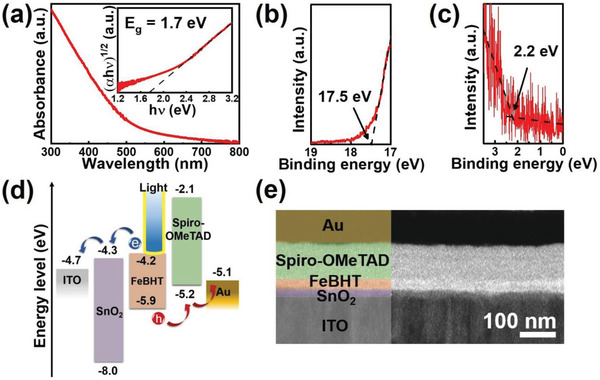
Device structure and energy band alignment of the FeBHT photodetector. a) Absorption spectrum of the FeBHT film. Inset: corresponding Tauc plot of the FeBHT film. UPS spectra of the FeBHT nanosheets. b) The secondary electron cutoff region and c) the valence band region. d) Energy level diagram of the FeBHT photodetector. e) Cross‐sectional TEM image of the FeBHT photodetector.

After verifying its electrical properties, we determined the intrinsic optical features of the FeBHT nanosheets by UV–vis absorption spectroscopy, as indicated in **Figure** **2a**. The FeBHT deposited on the substrate has a semitransparent appearance under ambient light (Figure 1b) because its visible light absorption spectrum is much weaker than that of the UV region. Moreover, the intense and broad UV absorption of the FeBHT film could be assigned to the *π*‐*π* ^*^ optical transition of the conjugated dithiolene skeleton.^[^ ^45^ ^]^ As a UV‐sensitized nanosheet, the energy band alignment of FeBHT extracted from a Tauc plot and ultraviolet photoelectron spectroscopy (UPS) can provide guidelines for the device structure designs of all solid‐state photodetectors. The Tauc plot (inset, Figure 2a) shows an indirect optical bandgap of 1.7 eV. Next, the full UPS spectrum (Figure S7, Supporting Information), enlarged secondary‐electron cutoff (Figure 2b), and enlarged valence‐band region (Figure 2c) of FeBHT are presented. The highest occupied molecular orbital (HOMO) level of FeBHT was −5.9 eV, which was obtained by extrapolating the onset of the cutoff binding energy to the background signal and the edge near the valence band region.^[^ ^46^ ^]^ Thus, the lowest unoccupied molecular orbital (LUMO) level was estimated as −4.2 eV by its HOMO‐LUMO bandgap of 1.7 eV. Associated with the band structure of FeBHT, we designed a well‐matched energy band alignment by introducing an ITO anode, a SnO_2_ electron transporter, a Spiro‐OMeTAD hole transporter and a Au cathode to build the FeBHT photodetector, as presented in Figure 2d. Among these components, the p‐type Spiro‐OMeTAD molecule, due to its excellent hole transport property as well as the suitable HOMO energy level,^[^ ^46^ ^]^ is a key part that can replace liquid electrolytes to fabricate all solid‐state devices. Figure 2e shows the corresponding cross‐sectional TEM image of a completed FeBHT photodetector. The FeBHT layer remains conformal and flat over a wide range, and the film thickness is ≈32 nm. In addition, the film thicknesses of the ITO cathode, SnO_2_ electron transporter, Spiro‐OMeTAD hole transporter and Au anode are ≈130, 17, 98, and 93 nm, respectively. Therefore, FeBHT was fully compatible with the fabrication process of the photodetector and may further enhance the UV response.

### 2.5 Photoresponse Characterization

**Figure 3:**
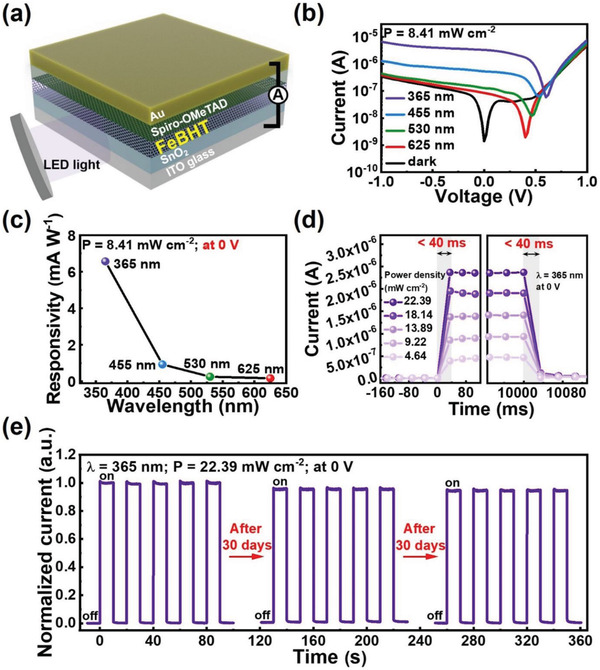
Electro‐optic characteristics of the FeBHT photodetector. a) Schematic structure of the FeBHT photodetector. b) *I–V* characteristics of the FeBHT photodetector under dark and light illumination at wavelengths ranging from 365 to 625 nm. c) Responsivities of the FeBHT photodetector as a function of wavelength under zero bias. d) Enlarged views of the time‐resolved photocurrent response near the rising (left) and decaying (right) edges of the FeBHT photodetectors with respect to various incident power intensities at 0 V under 365 nm illumination. e) Long‐term air stability of the nonencapsulated FeBHT photodetector. The device was first measured at 0 V under 365 nm illumination for 5 light on/off cycles. The other regular cycles were measured after storage in air for 30 days and 60 days.

**Figure** **3a** depicts a schematic of the FeBHT photodetector. The actual measurement setup of FeBHT photodetectors is shown in Figure S8a (Supporting Information). All measurements were performed under ambient conditions at room temperature by placing unencapsulated photodetectors in a holder without a vacuum system (Figure S8b, Supporting Information). The current–voltage (*I–V*) curves of the FeBHT photodetector as a function of incident light‐emitting diode (LED) wavelengths at a fixed power density (P) of 8.41 mW cm^−2^ are shown in Figure 3b. The *I–V* characteristics reveal the increase in photocurrents as the LED wavelength is reduced, which indicates that the FeBHT CONASH is suitable for operation as a light harvester in the low wavelength region. It is worth noting that the asymmetric *I–V* characteristics observed in the dark suggest that a p‐n junction is formed in the FeBHT photodetector. Moreover, after LED illumination, the open‐circuit voltage (*V*_OC_) was generated, which shows a typical photovoltaic effect^[^ ^35^, ^36^ ^]^ (that is, it allows operation in self‐powered mode with zero bias). The *V*_OC_ increases as the LED wavelength decreases, and this trend is consistent with that observed in the absorption spectrum (Figure 2a). This phenomenon verifies that the FeBHT photodetectors exhibited an excellent self‐powered UV light response, yielding a maximum *V*_OC_ of 0.60 V and a short‐circuit current (*J*_SC_) of 3.32 µA under 365 nm light illumination. In contrast, the photovoltaic effects of the devices without FeBHT were significantly weaker (Figure S9, Supporting Information). This comparison directly indicated that the FeBHT CONASH has photosensitive characteristics, especially in the low wavelength region. To precisely evaluate the performance of the FeBHT self‐powered UV photodetector, we calculated the responsivity R as a function of the wavelength with the equation R = I_ph_/P, where I_ph_ is the photocurrent density.^[^ ^47^, ^48^ ^]^ As presented in Figure 3c, under 365 nm LED illumination at a P of 8.41 mW cm^−2^, a promising spectral responsivity of 6.57 mA W^−1^, a specific detectivity of 3.13 × 10^11^ Jones and an external quantum efficiency of 2.23% were found. This spectral responsivity value is 1.6 times higher than that of the Fe_3_(THT)_2_(NH_4_)_3_ photodetector (the first solid‐state photodetector made of a CONASH‐based photoabsorber).^[^ ^19^ ^]^ Moreover, the self‐powered photodetector using the FeBHT‐based photoharvester has shown a comparable efficiency to that using P3HT, CH_3_NH_3_PbI_3_ perovskite, ZnO, and TiO_2_, as shown in Table S1 (Supporting Information). In addition, according to our previous extraction formula,^[^ ^14^ ^]^ the best quantum efficiency *φ* of the FeBHT photodetector is 5.94% under 365 nm radiation with an intensity of 8.41 mW cm^−2^, which is more than twice that of a bis(dipyrrin)zinc(II) photoelectrochemical cell comprising a porphyrin‐dipyrrin hybrid ligand (which is the most efficient photoelectrochemical cell containing a CONASH).^[^ ^15^ ^]^ This observation opens up the possibility of solid‐state CONASH photodetectors being state‐of‐the‐art devices that can improve the performance to a level higher than traditional liquid‐state photoelectric conversion systems.

### 2.6 Photoresponse Speed and Long‐term Device Stability

For a deeper understanding of the photoswitching properties of the FeBHT self‐powered UV photodetector, we measured the light‐response curves when the incident 365 nm LED was turned on and off once at various power densities (Figure S10, Supporting Information). Figure 3d presents magnified views of the temporal response. All the rise/decay times are observed to be less than 40 ms, which directly proves that regardless of how low the P value of the incident light is, excitons could be rapidly generated inside the FeBHT layer, quickly separated/transported through the photodetector, and effectively collected on electrodes.^[^ ^49^ ^]^ Moreover, expanded photoswitching cycles can be used to further explore the operational stability of the FeBHT device. As shown in Figure 3e, the recorded photocurrents of the unpackaged photodetector are almost constant even after multiple cycles under 365 nm illumination in air, implying that the organometallic FeBHT nanosheet can effectively avoid damage from high‐energy UV light and air through the aforementioned material engineering approach. Astonishingly, superior long‐term air stability was reported in an FeBHT self‐powered photodetector without any encapsulation. After placing the device in an ambient environment for 60 days, its photocurrent maintains 94% of the original value under 365 nm illumination (Figure 3e) and shows no obvious drop under 455 nm illumination (Figure S11, Supporting Information). To the best of our knowledge, this is the best long‐term air stability result reported so far for self‐powered photodetectors incorporating organic–inorganic light harvesters, thereby leading to potential practical applications of the FeBHT photodetector. For practical use, it is necessary to consider further improving the performance of the FeBHT photodetector. Film quality is the main issue to be solved. Based on our observation, the lower bonding rate between organic molecules and inorganic ions can produce high‐quality CONASH film. The lower concentration of precursors and lower synthesis temperature may effectively promote the film quality and thus obtain enhanced crystallinity. Moreover, the performance of the photodetector will also be improved.

### 2.7 FeBHT Oxygen Sensitivity Simulation

**Figure 4:**
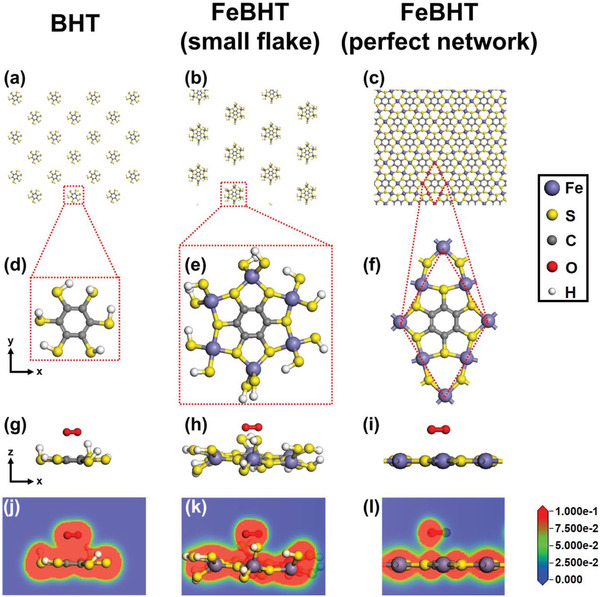
Insights from simulations of the air stabilities of FeBHT CONASHs. Schematic illustration of the chemical structures of a) BHT, b) small flake FeBHT, and c) perfect network FeBHT complex nanosheets. Top view of unit cell structures of d) BHT, e) small flake FeBHT, and f) perfect network FeBHT. DFT calculation of oxygen molecule adsorption onto the surface of g) BHT, h) small flake FeBHT, and i) the FeBHT with the perfect network. Total charge density cross‐sections of j) BHT, k) small flake FeBHT, and l) perfect network FeBHT after the adsorption of an oxygen molecule.

The origin of the BHT‐based CONASH instability in air was mainly attributed to the fact that the thiol groups of the redox‐active BHT moieties are easily oxidized.^[^ ^29^ ^]^ In this work, we used Fe(NH_4_)_2_(SO_4_)_2_ salt to decelerate the coordinated reaction between the Fe^2+^ ions and BHT molecules (Figure 1a), thus producing a FeBHT film with a large flake size (Figure 1b, right panel). This means that a greater proportion of thiol groups could bond with Fe^2+^ ions, thus preventing BHT from making direct contact with oxygen. To correlate the relation between this material engineering approach and oxidation resistance, we further conducted DFT simulations to illustrate the effect of BHT‐related materials with various configurations on the oxygen adsorption ability. The different oxygen adsorption energies of pristine BHT (hereafter called “BHT,” **Figure** **4a**), the smallest fragment of FeBHT (hereafter called “small flake,” Figure 4b) and FeBHT with a 2D periodic lattice (hereafter called “perfect network” in a hexagonal cell with *a* = *b* = 8.41 Å, Figure 4c) were calculated. Figure 4d–f shows the unit cells corresponding to Figure 4a–c, respectively. To directly compare the oxygen adsorption ability of the small flake and BHT, the terminal position of the smallest fragment of FeBHT was placed with the hydrogen atom. Figure 4g–i shows the side views of Figure 4d–f, respectively, and then an oxygen molecule was placed above these side‐viewed unit cells to understand the interactions between them. Cross sections of the charge densities of the BHT/O_2_, small flake/O_2_ and perfect network/O_2_ are shown in Figure 4j–l. The oxygen adsorption energy (*E*_ad_) between the BHT‐related materials and oxygen is defined as *E*_ad_ = *E*_total_ – *E*_BHT‐related materials_ – *E*_oxygen_, where *E*_total_ is the total energy of the oxygen molecules adsorbed on BHT‐related materials, *E*_BHT‐related materials_ is the total energy of the BHT‐related materials and *E*_oxygen_ is the total energy of an oxygen molecule.^[^ ^50^ ^]^ The calculated *E*_ad_ values for the BHT/O_2_, small flake/O_2_ and perfect network/O_2_ are −1.01, −0.82, and −0.74 eV, respectively. The remarkably increased *E*_ad_ values in both systems for the small flake/O_2_ and perfect network/O_2_ demonstrate that the FeBHT CONASHs have a better oxygen‐repelling nature than pristine BHT because their thiol groups are already protected by iron(II). Hence, after introducing metal ions with a low dissociation rate into the synthesis processes, the precursor‐to‐CONASH reaction can be promoted to increase the possibility of bonding between BHT and metal ions, leading to anaerobic properties. In addition, the *E*_ad_ value of the perfect network/O_2_ system was higher than that of the small flake/O_2_ system. That is, the FeBHT with a larger flake has more difficulty adsorbing oxygen onto its surface, which may be attributed to its excellent steric hindrance effect. As a result, the oxygen‐repelling FeBHT nanosheet could be synthesized via a reasonable material design, thus enabling the fabrication of air‐stable FeBHT self‐powered photodetectors.

## 3 Discussion

In summary, an organometallic 2D CONASH containing the FeBHT complex motif was presented as a UV absorber for the self‐powered photodetector. By introducing high conductive BHT ligand and using Fe(NH_4_)_2_(SO_4_)_2_ salt to slow down the liquid/liquid interfacial reaction rate, the synthesized FeBHT film can obtain high‐aspect‐ratio features with good crystallinity, resulting in high electrical conductivity and excellent oxidation resistance. As a result, the prototype FeBHT self‐powered photodetector had a high responsivity of 6.57 mA W^−1^, a specific detectivity of 3.13 × 10^11^ Jones, an external quantum efficiency of 2.23%, short response times (<40 ms) and substantially improved long‐term stability (>94% of its initial photocurrent after 60 days of aging) under 365 nm illumination in air without any encapsulation. These results not only show the high performance of the CONASH‐based self‐powered photodetector but also suggest 2D CONASH design strategies for the practical application of optoelectronic devices.

## 4 Experimental Section

### FeBHT Photodetector Fabrication

An electron‐transport SnO_2_ suspension (Alfa Aesar, 15% in a H_2_O colloidal dispersion) was diluted with deionized water to 2.67%. The SnO_2_ solution was then spin‐coated onto ITO glass at 3000 rpm for 30 s. The ITO/SnO_2_ substrate was heated at 150 °C for 30 min in air and then placed in a nitrogen‐filled glovebox. The ITO/SnO_2_ substrate was transferred to a container (60 mL, *ϕ* = 44 mm) made of perfluoroalkoxy alkane (PFA) in advance. The benzenehexathiol (BHT) molecule was synthesized according to a previous report.^[^ ^51^ ^]^ The BHT solution [1.35 mg of BHT was dissolved in 15 mL of degassed dichloromethane (DCM)] was added to the PFA‐based container first, and then 15 mL of deionized water was added on top of the BHT solution. Finally, the iron(II) solution [11.8 mg of ammonium iron(II) sulfate hexahydrate (Fe(NH_4_)_2_(SO_4_)_2_·6H_2_O, Sigma‐Aldrich, 99%) and 0.625 mg of sodium bromide (NaBr, FUJIFILM Wako Chemicals, 99.9%) were dissolved in 15 mL of degassed deionized water] was injected into the top of the solution. After 2 days, all of the solution was removed to obtain the FeBHT‐covered ITO/SnO_2_ substrate. The ITO/SnO_2_/FeBHT substrate was then washed thoroughly with ethanol to remove any residual impurities. After that, the ITO/SnO_2_/FeBHT substrate was naturally dried in air. Next, a hole‐transport solution was prepared by combining 1 mL of a 72.3 mg mL^−1^ solution of 2,2′,7,7′‐tetrakis‐(*N*,*N*‐di‐*p*‐methoxyphenylamine)9,9′‐spirobifluorene (Spiro‐OMeTAD, Borun Chemicals, 99.7%) in chlorobenzene (CB), 28.8 µL of 4‐*tert*‐butylpyridine (TBP, Sigma‐Aldrich, 96%), and 17.5 µL of a 520 mg mL^−1^ lithium bis(trifluoromethylsulfonyl)imide (LiTFSI, Sigma‐Aldrich, 99.9%) stock solution in acetonitrile (ACN). The hole transport layer was spin‐coated from the Spiro‐OMeTAD solution at 2000 rpm for 30 s, followed by deposition of an 100 nm gold electrode by thermal evaporation.

### Characterization and Measurements

X‐ray photoelectron spectroscopy (XPS) measurements were recorded using a VG Scientific SIGMA PROBE. XPS survey scans were collected to identify the overall surface composition using a monochromatic Al K*α* X‐ray source (1486.6 eV). All of the XPS spectra were calibrated using the 284.6 eV peak (that is, the C 1s core level). Absorption spectra were obtained using a LAMBDA 365 UV/vis spectrometer (PerkinElmer). Ultraviolet photoelectron spectroscopy (UPS) measurements were conducted with a VG Scientific SIGMA PROBE with a He I (21.2 eV) discharge lamp. The transmission electron microscopy (TEM) specimens of the FeBHT photodetector were prepared in a focused ion beam (FIB) system (FEI Helios 600i). TEM images and energy‐dispersive X‐ray spectroscopy (EDS) spectra in a scanning transmission electron microscopy (STEM) system of the FeBHT photodetector and the FeBHT nanosheet were obtained with TEM at 200 kV (JEOL 2010F). The current–voltage (*I–V*) characteristics and photoresponse behaviors of the FeBHT photodetectors were studied in a customized cell (Ossila, Inc.) by using a Keithley 2401 source meter under LED light illumination applied by a Mightex WFC‐series multiwavelength fiber‐coupled LED source. The light intensity and the light on/off cycle times were directly tuned by the controller program of the LED light source. All measurements to determine the photodetector performance were conducted under ambient conditions.

### Theoretical Calculations

Calculations based on density functional theory (DFT) with the Cambridge Serial Total Energy Package (CASTEP) program were performed to investigate why FeBHT photodetectors have high air stability. The Perdew‐Burke‐Ernzerhof (PBE) generalized gradient approximation (GGA) was used for the exchange correlation functional. The plane‐wave basis sets were used, and the energy cutoff was set to 340 eV. The ion–electron interaction was modeled by the ultrasoft pseudopotential. The total energies were optimized until the force per atom was less than 0.03 eV Å^−1^. For an accurate description, the electronic structures of oxygen or water located on FeBHT or BHT were calculated by DFT with an empirical correction dispersion (DFT‐D) due to the existence of van der Waals interactions^[^ ^52^ ^]^ between oxygen/water and FeBHT/BHT. The BHT and FeBHT calculations using the gamma *k*‐point and the 3 × 3 × 1 Monkhorst‐Pack *k*‐point were performed, respectively.

## Conflict of Interest

The authors declare no conflict of interest.

## Supporting information

Supporting Information

## Data Availability

Research data are not shared.
