# Supplementary material for: Two‐Dimensional Bis(dithiolene)iron(II) Self‐Powered UV Photodetectors with Ultrahigh Air Stability
Source: Adv Sci (Weinh). 2021 May 19;8(14):2100564. doi: 10.1002/advs.202100564 (PMC8292878; doi:10.1002/advs.202100564)
Supplement: Supplementary file 1 — Supporting Information [file ADVS-8-2100564-s001.pdf]

© 2021 Wiley-VCH GmbH

ADVANCED SCIENCE Open Access

Supporting Information

for *Adv. Sci.,* DOI: 10.1002/advs.202100564

Two-Dimensional Bis(dithiolene)iron(II) Self-Powered UV Photodetectors with Ultrahigh Air Stability

Ying-Chiao Wang, Chun-Hao Chiang, Chi-Ming Chang, Hiroaki Maeda, Naoya Fukui, I-Ta Wang, Cheng-Yen Wen, Kuan-Cheng Lu, Shao-Ku Huang, Wen-Bin Jian, Chun-Wei Chen, Kazuhito Tsukagoshi,* and Hiroshi Nishihara**

Supporting Information

Two-Dimensional Bis(dithiolene)iron(II) Self-Powered UV Photodetectors with Ultrahigh Air Stability

Ying-Chiao Wang,# Chun-Hao Chiang,# Chi-Ming Chang, Hiroaki Maeda, Naoya Fukui, I-Ta Wang, Cheng-Yen Wen, Kuan-Cheng Lu, Shao-Ku Huang, Wen-Bin Jian, Chun-Wei Chen, Kazuhito Tsukagoshi,* and Hiroshi Nishihara**

#These authors contributed equally.

Table S1. Performance comparison of self-powered photodetectors with those in the previous literature.

Device structure	Wavelength (nm)	Responsivity (mA W^{-1})	Ref.
In-Ga/Si/$CuGaTe_2$/Ag	1064	114	[1]
ITO/SnO_2/$Cs_2AgBiBr_6$/Au	350	110	[2]
FTO/TiO_2/Ag NW	350	32.5	[3]
In/p-Si/Ga_2O_3/PEDOT:PSS/Ti/Au	255	29	[4]
**ITO/SnO_2/FeBHT/Spiro-OMeTAD/Au**	**365**	**6.57**	**This work**
Ag/ITO/ZnO NW/$CuCrO_2$/Ag	395	5.87	[5]
ITO/$CH_3NH_3PbI_3$/Ag	808	1.42	[6]
In/ZnO nanofiber/In	360	1	[7]
Ti/TiO_2/P3HT/CNT	350	0.25	[8]

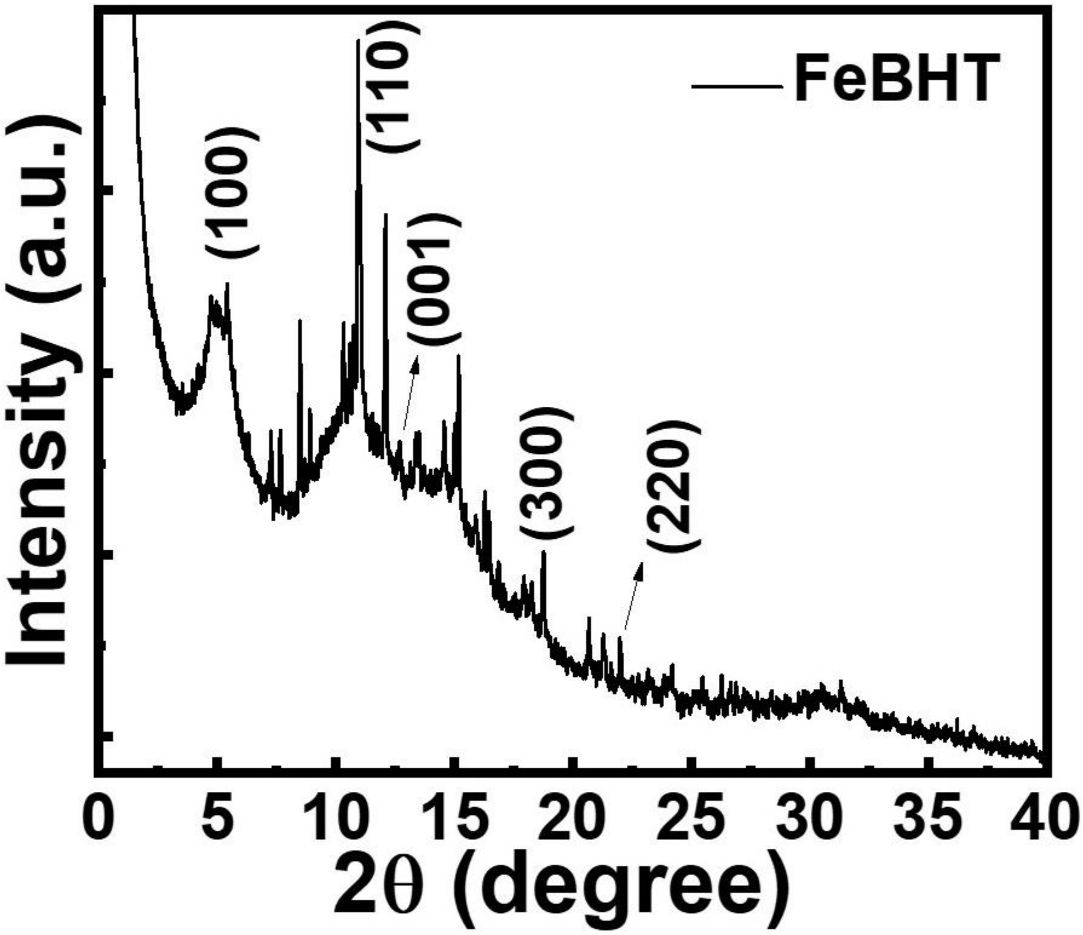

Figure S1. Powder XRD patterns of the FeBHT CONASH.

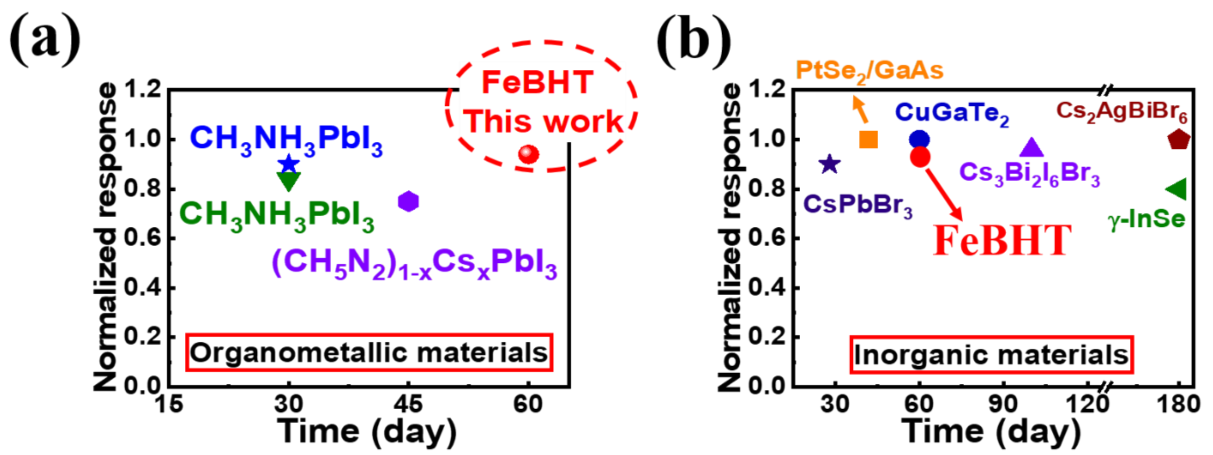

Figure S2. The long-term air stability comparison of the self-powered photodetectors with the reported (a) organometallic and (b) all inorganic systems.

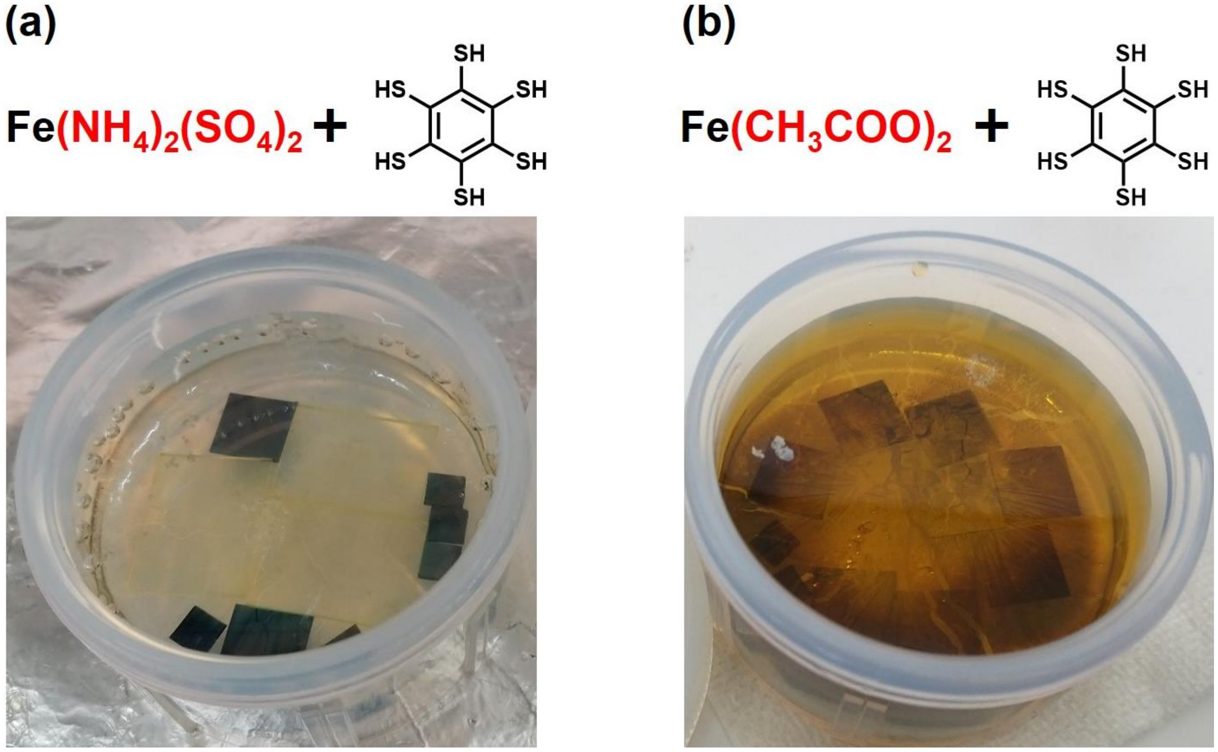

Figure S3. Film- forming properties of the FeBHT nanosheets prepared with different iron(II) salts. Synthesis of FeBHT nanosheets with the BHT molecule and various iron(II) precursors: a) $Fe(NH_4)_2(SO_4)_2$ and b) $Fe(CH_3COO)_2$. The photographs below the individual chemical reaction formulas show the as-prepared FeBHT films prepared with different iron sources.

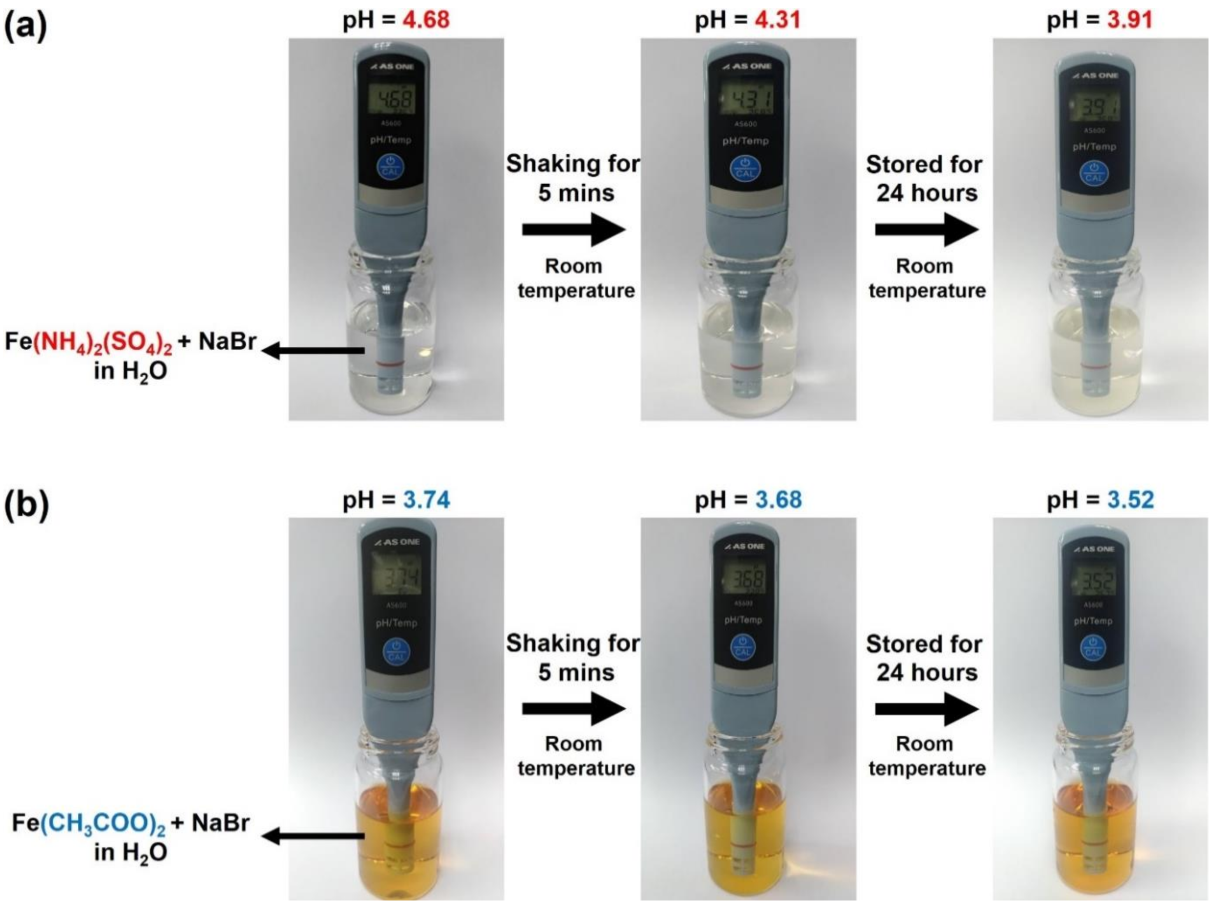

Figure S4. Dissociation rates of iron(II) salts. Observed pH shift in aqueous solutions based on various iron(II) precursors: a) $Fe(NH_4)_2(SO_4)_2$ and b) $Fe(CH_3COO)_2$. The observations were separated into the following three stages: 1. The as-prepared aqueous solution (left); 2. after shaking for 5 minutes (center); 3. after storing for 24 hours (right).

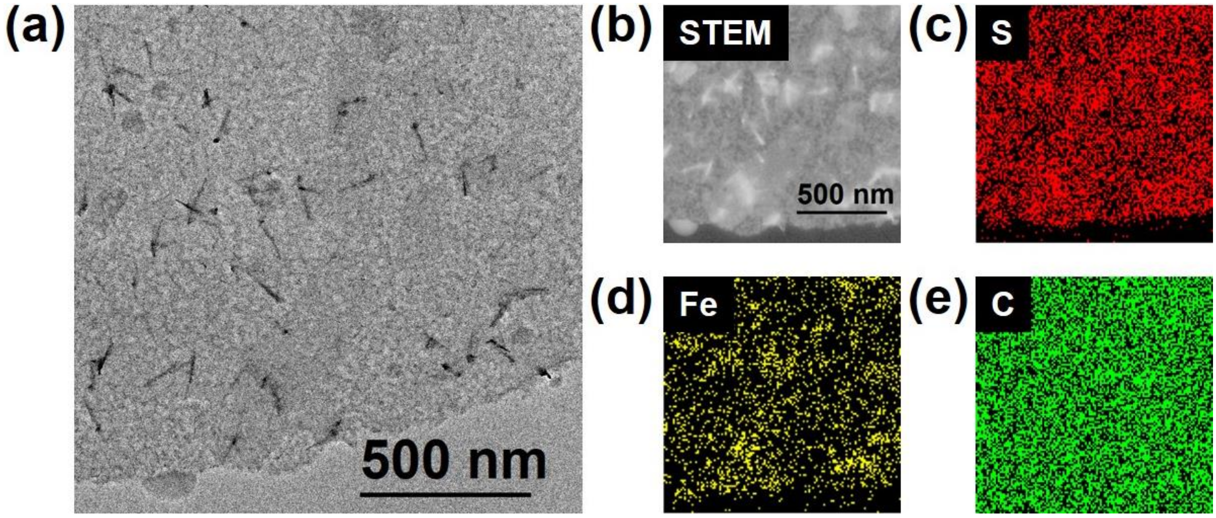

Figure S5. Surface observation of the as-synthesized FeBHT nanosheet. Top-view a) TEM and b) STEM HAADF images of the FeBHT nanosheet. The corresponding elemental maps of c) S, d) Fe and e) C.

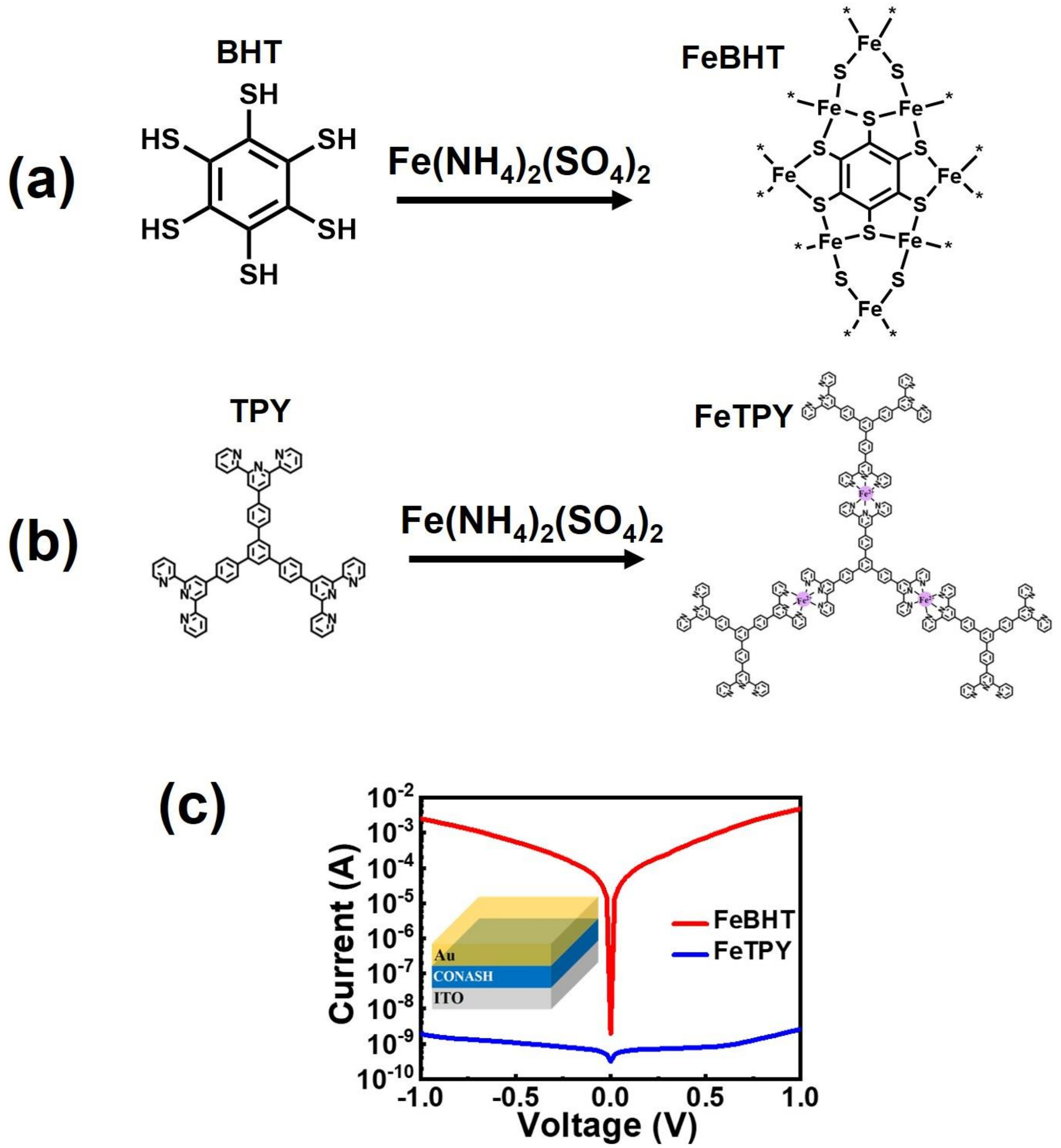

Figure S6. The influence of organic ligands on the electrical conductivities of CONASHs. The formation routes of a) the FeBHT complex CONASH and b) the FeTPY complex CONASH. c) The corresponding current- voltage characteristics of Fe-based CONASHs. The inset displays the structure of the CONASH-based device.

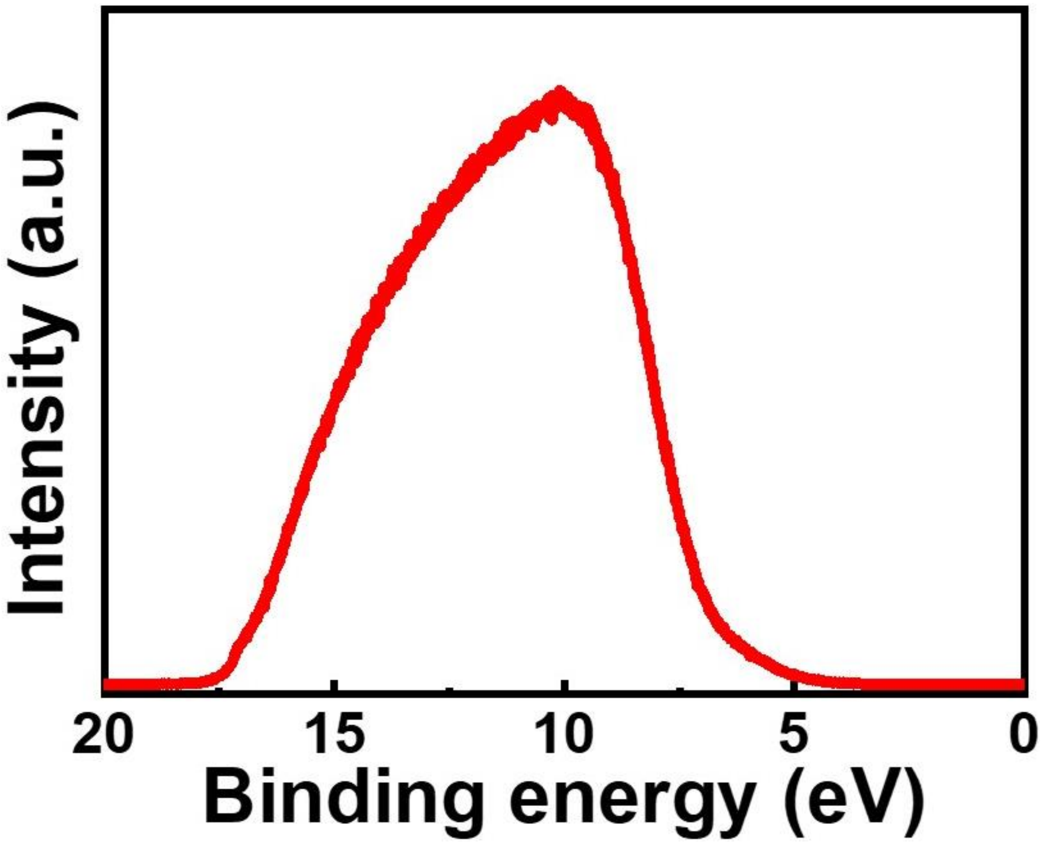

Figure S7. UPS spectrum of the FeBHT nanosheet. The full UPS spectrum is presented.

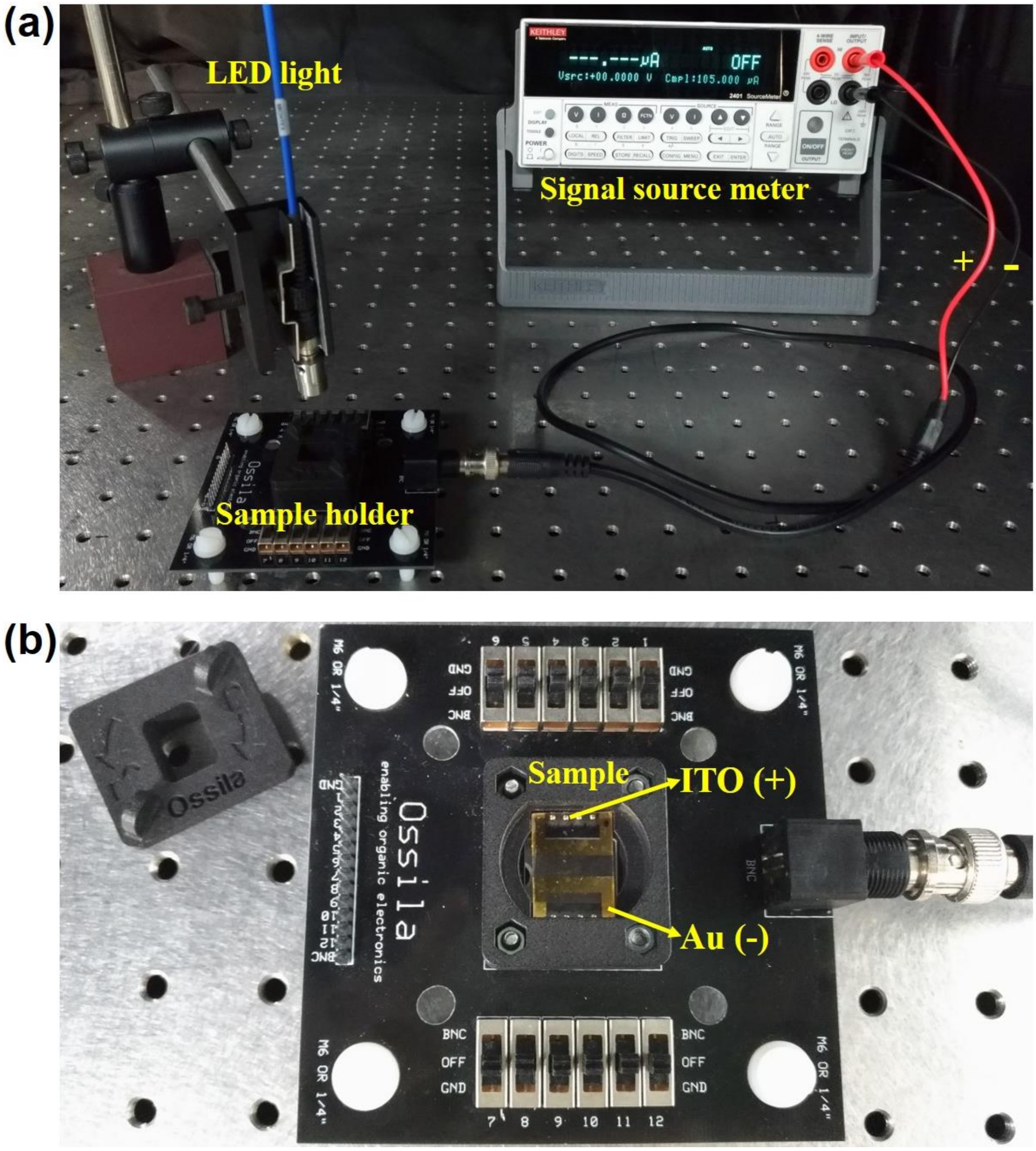

Figure S8. Measurement setup of photodetectors. a) Photographs of the experimental setup and b) the corresponding FeBHT photodetector and its holder for taking measurements. All measurements were carried out at room temperature in air. All devices were unpackaged.

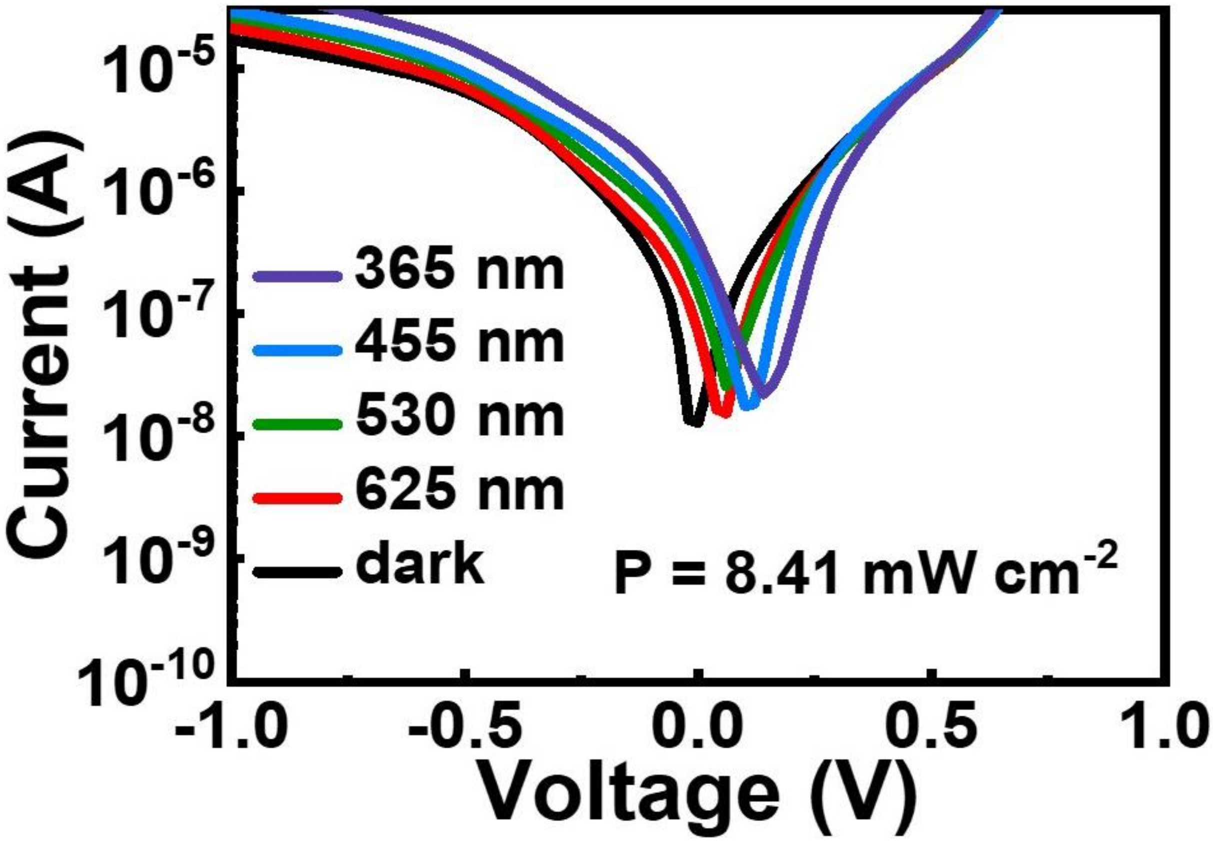

Figure S9. Electro-optic characteristics of the optoelectronic device without CONASHs. *I*-*V* characteristics of the ITO/SnO_2/Spiro-OMeTAD/Au device measured under dark and light illumination at wavelengths ranging from 365 to 625 nm.

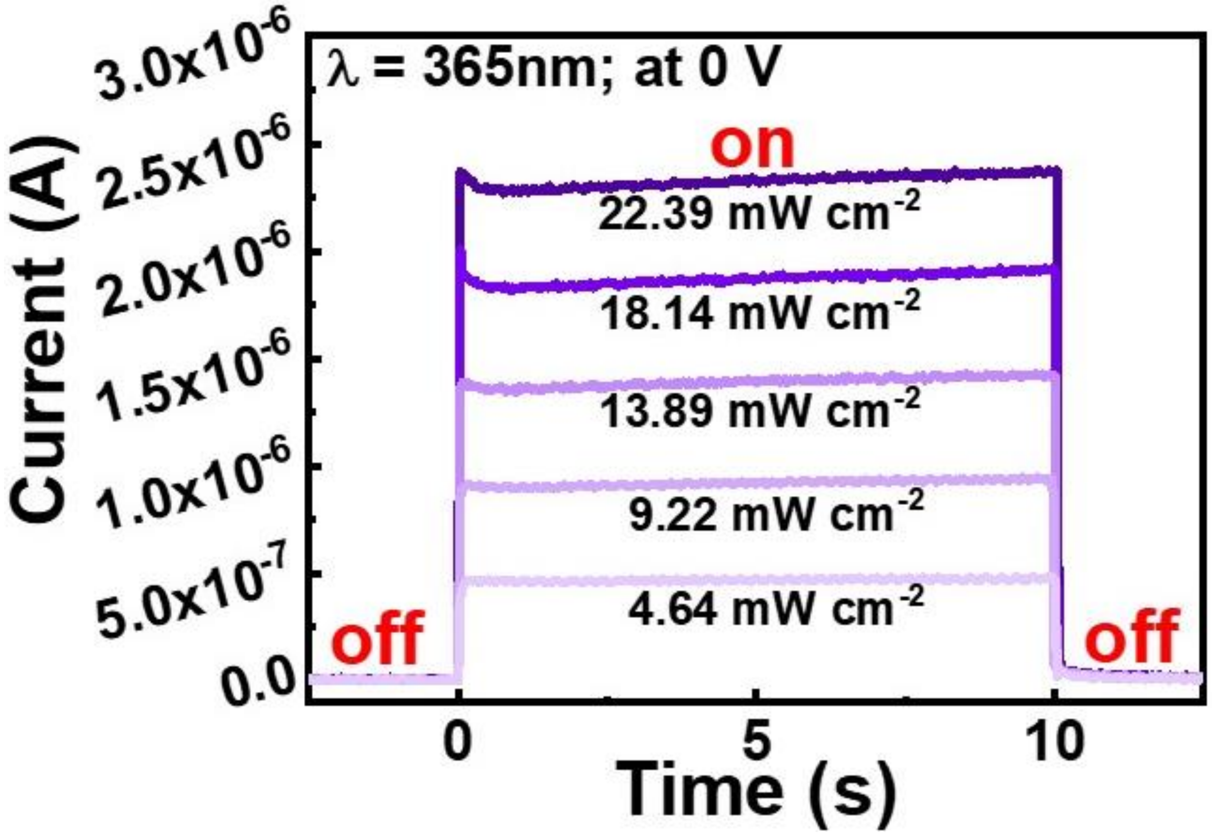

Figure S10. Power-dependent photoresponses of the FeBHT photodetector. Time response characterizations of FeBHT photodetectors under a light on/off cycle at a fixed bias of 0 V under 365 nm illumination.

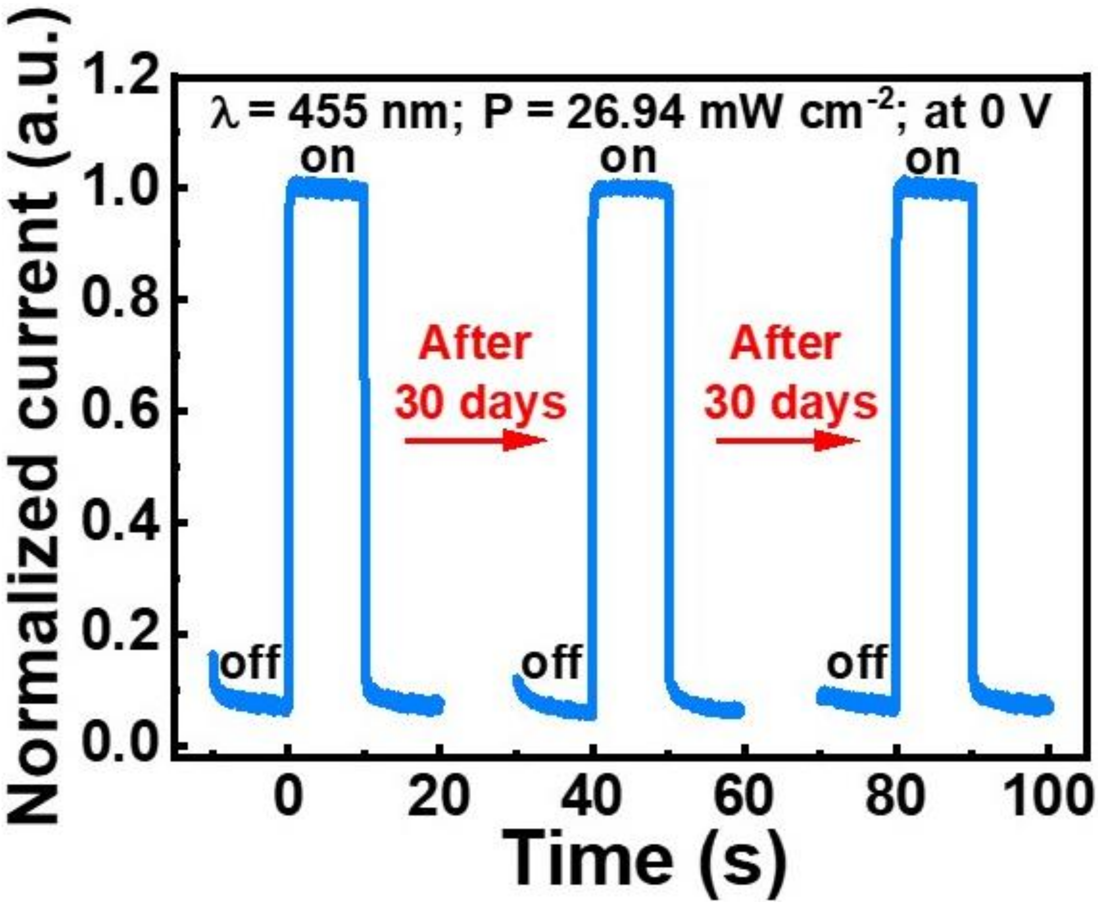

Figure S11. Air-stable self-powered visible photodetector. Long-term air stability of the nonencapsulated FeBHT photodetector. The device was first measured at 0 V under 455 nm illumination for 5 light on/off cycles. The other regular cycles were measured after the device was stored in air for 30 days and 60 days.

Working principle of the FeBHT self-powered photodetector: Based on its self-driving photovoltaic effect, the self-powered photodetector can work without an external power supply. According to the charge separation characteristics at the interfaces, the self-powered photodetector can be divided into the following three categories: photoelectrochemical device, Schottky junction and PN junction (or PIN junction). Among them, the FeBHT self-powered photodetector belongs to the PIN structure (that is, p-type Spiro-OMeTAD/intrinsic light absorbing FeBHT layer/n-type SnO_2). Under dark without bias applied, the Fermi level reaches thermal equilibrium across the entire FeBHT self-powered photodetector. Since the Spiro-OMeTAD layer and the SnO_2 layer exhibit a high work function and low work function, respectively, the thermal equilibrium will generate a built-in potential. Due to the intrinsic properties of the FeBHT CONASH, the bands (Spiro-OMeTAD, SnO_2 and FeBHT) are inclined along the entire FeBHT layer, with the electrical field locating at the intrinsic area. In the model, contact selectivity is generated by an electric field, which pushes holes and electrons to Spiro-OMeTAD and SnO_2, respectively. At the same time, the drift currents play the key roles in carrier separation and collection. Under irradiating under the open-circuit condition, the splitting of the Fermi levels will produce a flat band situation. In this case, the electrical field disappears. As a result, the collection driving force is cancelled and the photocurrent is eliminated. Therefore, the open-circuit condition is determined by the work functions of the P and N materials. In the PIN structure, after the FeBHT absorbs UV light, the photogenerated excitons inside the FeBHT layer can be separated and transferred across the transporters. Finally, we can collect these photogenerated free carriers from electrodes. Moreover, several key parameters can be used to evaluate the performance of the self-powered photodetector, as follows: the light on/light off ratio, photoresponsivity (defined as the current passing through the photodetector per working area per effective power of light), detectivity (defined as the limit of light intensity that the device can detect) and light response

time (defined as the time required for the photocurrent to rise and decay during the on/off cycles of light illumination).[9,10]

Calculation formula: To quantify the photodetector performance, there are several parameters as the key indicators needed to be considered.[11-13] The value of spectral responsivity (R) is calculated via the equation (1):

$$R = I_{ph}/P, \tag{1}$$

where I_{ph} is the photocurrent density (in A cm^{-2}), and P is power density of the illumination light source, which is directly obtained via optical power meter (in W cm^{-2}).[11,12] The specific detectivity (D^*) is expressed by the equation (2):

$$D^* = R/(2qI_d)^{1/2}, \tag{2}$$

where q is the elementary charge (1.60×10^{-19} C), and I_d is the dark current density of the device (in A cm^{-2}).[11,12] For the conversion efficiency, the external quantum efficiency (EQE) and quantum yield (φ) are defined by the equation (3) and (4), respectively:

$$EQE = Rhc/q\lambda \tag{3}$$

$$\varphi = Rhc/q\lambda(1\text{-}10^{-A}), \tag{4}$$

where h is the Planck constant (6.63×10^{-34} J s), c is the velocity of light (3.00×10^{8} m s^{-1}), λ is the wavelength of incident light (in m), and A is the absorbance at the incident light wavelength.[11-13]

Supplementary References

[1] L. Wang, Z. Li, M. Li, S. Li, Y. Lu, N. Qi, J. Zhang, C. Xie, C. Wu, L.-B. Luo, *ACS Appl. Mater. Interfaces* **2020**, 12, 21845.

[2] C. Wu, B. Du, W. Luo, Y. Liu, T. Li, D. Wang, X. Guo, H. Ting, Z. Fang, S. Wang, Z. Chen, Y. Chen, L. Xiao, *Adv. Optical Mater.* **2018**, 6, 1800811.

[3] H. Fang, C. Zheng, L. Wu, Y. Li, J. Cai, M. Hu, X. Fang, R. Ma, Q. Wang, H. Wang, *Adv. Funct. Mater.* **2019**, 29, 1809013.

[4] D. Zhang, W. Zheng, R. Lin, Y. Li, F. Huang, *Adv. Funct. Mater.* **2019**, 29, 1900935.

[5] T. Cossuet, J. Resende, L. Rapenne, O. Chaix- Pluchery, C. Jiménez, G. Renou, A. J. Pearson, R. L. Z. Hoye, D. Blanc- Pelissier, N. D. Nguyen, E. Appert, D. Muñoz- Rojas, V. Consonni, J.- L. Deschanvres, *Adv. Funct. Mater.* **2018**, 28, 1803142.

[6] C. Perumal Veeramalai, S. Yang, R. Zhi, M. Sulaman, M. I. Saleem, Y. Cui, Y. Tang, Y. Jiang, L. Tang, B. Zou, *Adv. Optical Mater.* **2020**, 8, 2000215.

[7] Y. Ning, Z. Zhang, F. Teng, X. Fang, *Small* **2018**, 14, 1703754.

[8] L. Zheng, X. Deng, Y. Wang, J. Chen, X. Fang, L. Wang, X. Shi, H. Zheng, *Adv. Funct. Mater.* **2020**, 30, 2001604.

[9] W. Tian, Y. Wang, L. Chen and L. Li, *Small* **2017**, *13*, 1701848.

[10] I. Mora-Seró, *Joule* **2018**, *2*, 583-593.

[11] Y. Wang, R. Fullon, M. Acerce, C. E. Petoukhoff, J. Yang, C. Chen, S. Du, S. K. Lai, S. P. Lau, D. Voiry, D. O'Carroll, G. Gupta, A. D. Mohite, S. Zhang, H. Zhou, M. Chhowalla, *Adv. Mater.* **2017**, *29*, 1603995.

[12] M. Yang, J. Wang, Y. Zhao, L. He, C. Ji, X. Liu, H. Zhou, Z. Wu, X. Wang, Y. Jiang, *ACS Nano* **2018**, *13*, 755.

[13] R. Sakamoto, K. Hoshiko, Q. Liu, T. Yagi, T. Nagayama, S. Kusaka, M. Tsuchiya, Y. Kitagawa, W.-Y. Wong, H. Nishihara, *Nat. Commun.* **2015**, *6*, 6713.
